# Efficacy of lotilaner (Credelio™), a novel oral isoxazoline against naturally occurring mange mite infestations in dogs caused by *Demodex* spp.

**DOI:** 10.1186/s13071-017-2472-2

**Published:** 2017-11-01

**Authors:** Daniel E. Snyder, Scott Wiseman, Julian E. Liebenberg

**Affiliations:** 1 Elanco Animal Health, Research and Development, 2500 Innovation Way, Greenfield, IN 46140 USA; 2 Elanco Animal Health, Basingstoke, Hampshire RG24 9NL UK; 3 ClinVet International (pty) Ltd, Uitsigweg, Bainsvlei, 9338 Bloemfontein, Republic of South Africa

**Keywords:** Credelio™, *Demodex* spp., Demodicosis, Dog, Lotilaner, Mange, Oral

## Abstract

**Background:**

The oral systemic efficacy of lotilaner (Credelio™, Elanco) was evaluated against *Demodex* spp. in naturally infested dogs with generalized demodicosis.

**Methods:**

In this study, 10 dogs with clinical signs of generalized demodicosis and positive for *Demodex* spp. mites based on skin scrapings were assigned to a single group orally treated with lotilaner (minimum dose of 20 mg/kg) on Days 0, 28 and 56.

**Results:**

For lotilaner-treated dogs, pre-treatment mite counts based on skin scrapings performed at five different sites were reduced by > 99.9% (*P* < 0.0001) up to 56 days after the first and second monthly doses. No live mites were detected after Day 56 out to and including Day 84 post-treatment for 100% efficacy of each dog’s *Demodex* mite infestation. Nine of 10 dogs were 100% mite-free from Day 28 (first evaluation) through Day 84 (end of study) and live mites were only found once on one dog (Day 56) following treatment with lotilaner. All dogs in the lotilaner-treated group showed marked improvement in the clinical signs of demodicosis and there were no drug associated adverse events. A marked improvement in hair re-growth was observed in all the dogs from 6 weeks following initiation of treatment.

**Conclusions:**

In this study lotilaner administered at a minimum oral dose of 20 mg/kg was highly effective in reducing and eliminating live mite counts in dogs with natural infestations of *Demodex* spp.

**Electronic supplementary material:**

The online version of this article (10.1186/s13071-017-2472-2) contains supplementary material, which is available to authorized users.

## Background

Canine demodicosis is a parasitic skin disease characterized by an excessive increase of *Demodex* spp. mites within the pilosebaceous glands [1]. Three species of *Demodex* mites are implicated, with *Demodex canis* being the most common [2]. Canine demodicosis is classified as localized or generalized according to the extent of the disease. Localized demodicosis is a benign disease and most cases resolve spontaneously within 6 to 8 weeks [1]. Demodicosis is considered generalized when five or more areas of localized disease are observed, pododemodicosis is observed on two or more feet, or when an entire body region is involved. Demodicosis can also be categorized as either juvenile (dogs up to 18 months of age), adult onset (dogs generally older than 4 years of age with no previous history of disease), or chronic generalized (persisting disease for at least 6 months) [2, 3].

Chronic generalized demodicosis is a frustrating and difficult skin disease to treat [4, 5]. In these dogs with long term chronic generalized demodicosis, the disease is unlikely to resolve without therapy [3]. Currently available therapeutic options involve daily, weekly, bi-weekly or monthly treatments for periods of 3 months or more [1, 5, 6].

Lotilaner (Credelio™, Elanco) is a novel isoxazoline with demonstrated potent month long activity against ticks and fleas [7, 8] following oral administration. The study as reported herein was conducted to evaluate the efficacy and safety of lotilaner against natural infestations of *Demodex* spp. in dogs with generalized demodicosis at the minimum dose of 20 mg/kg as proposed for monthly administration for the treatment and control of fleas and ticks.

## Methods

This study was conducted in the Republic of South Africa in compliance with Good Clinical Practice [9] and local animal welfare guidelines [10]. The study protocol was reviewed and approved by ClinVet and Elanco Institutional Animal Care and Use Committees.

### Animals and housing

Dogs were housed individually in cages under strict quarantine conditions during the entire study. No physical contact between dogs was possible, but dogs did have visual and auditory contact with conspecifics and housing conditions conformed to accepted animal welfare guidelines. Each dog was individually identified by an alphanumeric microchip implant. The dogs enrolled in this study had not been treated with an ectoparasiticide for at least 90 days and were in good health at the time of treatment except for clinical signs of alopecia and skin lesions associated with generalized demodicosis. Dogs were fed an appropriate maintenance ration of a commercial dry canine food for the duration of the study. Water was available *ad libitum*.

The study included 10 (7 male and 3 female), locally sourced, adult mongrel dogs ≥ 6 months of age and weighing from 9.59 to 17.65 kg. Dogs were enrolled in the study based on positive skin scrapings with natural *Demodex* infestations. All enrolled dogs exhibited clinical signs of generalized demodicosis including skin lesions such as alopecia, erythema, comedones, papules, pustules, casts, scales or crusts involving an entire body region or five or more localized lesions in discreet areas (each with a diameter > 2.5 cm), or pododemodicosis involving two or more feet, and the confirmed presence of live *Demodex* mites based on deep skin scrapings [11, 12]. Additionally, no dogs had received any short acting corticosteroids within 1 week or a long acting corticosteroid within 4 weeks of Day 0.

### General experimental design and methods

Dogs were acclimated to the study site conditions for 7 days prior to treatment. This study was conducted with no negative control group due to animal welfare concerns. Day 0 was defined as the day when all 10 dogs received their first treatment. A physical examination was performed on each dog by the attending veterinarian on Day -7 to determine health and suitability prior to inclusion in the study, and general health observations were made at least once daily throughout the study. Additionally, a clinical examination was conducted on each dog on Days -2, 14, 28, 42, 56, 70 and 84.

Starting on Day -7 all enrolled dogs received an appropriate antibiotic (as prescribed by the attending veterinarian) for pyoderma. Concurrently with the antibiotic therapy, all dogs also received brewer’s yeast daily to support their intestinal flora. On Days -7 and 27, biopsies were taken from each dog under sedation with a suitable sedative, and antimicrobial concomitant therapy was continued until receipt of these biopsy results. If, based on Day 27 biopsy results, no inflammatory cells or bacteria were present, antimicrobial therapy was discontinued. If, based on Day 27 biopsy results, there was still evidence of active infection, but less inflammatory cells and/or bacteria compared to the first skin biopsies, the antibiotic and brewer’s yeast originally used were continued for another 4 weeks. If clinical and cytological deterioration was detected based on Day 27 biopsy results, the antibacterial therapy and brewer’s yeast were continued with an alternative antibiotic as prescribed by the attending veterinarian.

Lotilaner was administered once on Days 0, 28, and 56 and was based on the weights of the dogs, rounded to the nearest 0.1 kg, recorded on the day preceding each treatment. Mite counts performed on dosing days were conducted prior to each dog being dosed with lotilaner. The body weights obtained during acclimatization were used for the Day 0 dosing and the body weight measured on the day prior to each subsequent scheduled dosing time point (Days 28 and 56) were used to determine the appropriate strength and number of lotilaner tablets that were administered at each dosing time point, respectively.

Oral dosing was conducted in the fed state. On the day prior to each scheduled dosing time point (Day -1, Day 27 and Day 55), each dog was offered only half of its daily ration of food. On each treatment day (Days 0, 28 and 56), 30 ± 5 min prior to each scheduled lotilaner treatment, each dog was offered a daily ration of wet canned food at the recommended rate. The time of feeding was recorded and at the time of scheduled treatment (± 5 min), the residual food was removed, weighed and recorded. At least 1/3 of the daily ration offered was required to be consumed by the dog prior to the administration of the lotilaner tablets. If the dog did not consume the wet food after 15 min (± 5 min), then the dog was manually fed by placing small amounts of the food into the back of the mouth and allowing the dog to swallow until the approximate half a tin of wet foot had been consumed and then treatment proceeded as scheduled. Tablets were orally administered by pilling to ensure accurate and complete dosing. Each dog was observed for several minutes post-dosing, and then at approximately 1 h intervals after dosing up to 4 h post-dosing to observe each dog for any signs of abnormal health.

To avoid any issues of contamination of dogs during mite counts and clinical examinations, study personnel changed protective clothing between dogs, utilized separate gloves and equipment with each dog, and cleaned the surface of the examination table used for scraping and/or mite counting.

All dogs were examined for the clinical signs of demodicosis (including photographic documentation) and skin scrapings/mite counts were performed on Days -2, 28, 42, 56, 70 and 84. If any dog demonstrated two consecutive negative (zero) live mite counts on or after Day 56, additional deep skin scraping at the time points listed above were stopped for animal welfare reasons due to the invasive nature of this procedure, but clinical assessments and photographic documentation were continued until Day 84 to fully assess the resolution of skin lesions. Mite infestations were evaluated using deep skin scrapings taken from five sites on each dog at each scheduled time point showing the most severe clinical evidence of an active mite infestation based on examination of visibly affected skin lesions. Scraped material was transferred to a slide, mixed with mineral oil and examined under a microscope using 40× or 100× magnification to count adult and immature mites. The clinical signs and the extent of demodectic lesions on each dog were assessed on the days during which scrapings are made and recorded on a standardized form. The following parameters were assessed for each dog and sketched on a silhouette (left and right hand side) of a dog: (i) body areas covered by casts, scales and crusts; (ii) body areas with hair loss (1, slight thinning of hair; 2, conspicuous hair loss; 3, no hair); and (iii) body areas with erythema. The clinical signs of generalized demodicosis were assessed as the percent of the body surface affected by skin lesions followed by the assignment of a clinical score to each of four parameters: (i) comedones, pustules and papules; (ii) casts, crusts and scales; (iii) alopecia; and (iv) erythema.

### Mite counts and efficacy evaluation

The primary assessment variable in this study was the percent decrease in mite counts (immature and adult live mites combined) as compared to baseline counts on days 28, 42, 56, 70 and 84, following monthly administration of the lotilaner.

Efficacy was calculated using geometric means with Abbott’s formula:

$$ \mathrm{Efficacy}\ \left(\%\right)=\left(\mathrm{Mpre}-\mathrm{Mpost}\right)/\mathrm{Mpre}\times 100 $$

where Mpre was the group mean number of pre-treatment mite counts, and Mpost the group mean number of post-treatment mite counts.

Where no count was available for a dog on or after day 56, because the dog had two successive zero counts at earlier time points, then zero was used in the assessment.

Additionally, a secondary efficacy assessment variable was the individual percentage decrease from the pre-administration mite count to the post-administration mite count in each dog on each assessment day and was calculated by:

Decrease % (individual) = (Pre-administration – Post-administration)/Pre-administration × 100

where Pre-administration is the pre-administration mite count of a dog, and Post-administration is the post-administration mite count of a dog.

The number of mites on each assessment day and percentage reduction in mite counts were tabulated, with the following descriptive statistics: mean, standard deviation (SD), geometric mean (GM), minimum and maximum. Additionally, another secondary efficacy assessment variable was the cure rate, defined as follows:

Cure rate (expressed as a percentage) = number of mite-free dogs after two consecutive post-treatment zero mite counts / total number of dogs with pre-treatment mite counts.

### Clinical signs

Data recorded during clinical assessments on casts, scales, crusts and area(s) of hair loss and erythema, were summarized by the investigator. Overall changes in clinical appearance were also documented by pre- and post-administration photographs for each dog. This photographic record was utilized to show the overall extent and resolution of demodectic lesions for each dog.

Furthermore, the number of dogs affected by erythema, casts, scales and crusts were described for the pre-administration and different post-administration assessment days. A semi-quantitative assessment of hair re-growth was also done and a score awarded to each dog on each of the different post-dosing assessment days.

A semi-quantitative assessment of hair re-growth was assigned a score of: (i) if body areas with hair re-growth 0–50% compared to that recorded during the pre-administration assessment; (ii) body areas with estimated hair re-growth > 50% ≤ 90% compared to that recorded during the pre-administration assessment; and (iii) body areas with estimated hair re-growth > 90% compared to that recorded during the pre-administration assessment.

The clinical signs were analyzed descriptively using frequency counts and percentages to assess the efficacy of the monthly lotilaner administrations.

### Statistical methods

The experimental unit was the individual dog. The WAAVP guidelines [13] and the EMEA guidelines [14] recommend a minimum of six subjects per group are used in studies assessing the efficacy of products against fleas and ticks. With as few as five dogs in the lotilaner treatment group, it was possible to detect a statistically significant drop (at the 5% type I error level) of > 90% in mite counts with greater than 90% power. The use of 10 dogs was considered appropriate to provide robust statistical and clinical outcomes in this single group study. Statistical testing was conducted at the 5% level of significance.

The effect of treatment was investigated on mite count for each dog was investigated by comparing pre-treatment baseline and post-treatment mite counts in an ANOVA model with time (pre- or post-treatment) and dog fixed effects. Separate models were fitted for Study Days 28, 42 and 56). Only one dog had skin scrapings performed on Days 70 and 84 so no statistical testing was possible.

#### Comparison of mite counts

The pre-treatment baseline and post-treatment mite counts for each dog were compared by an ANOVA model with time (pre- or post-treatment) and dog effects using SAS Version 9.3 TS Level 1 M2 (SAS Institute Inc., Cary, NC, USA). To assess the hypothesis that the observed percentage cure rate as calculated in this study was no different from a theoretical spontaneous self-cure rate of 50%, a statistical analysis using the exact test of a binomial proportion was performed.

#### Efficacy claim

Efficacy was claimed for lotilaner against mites if a ≥ 90% statistically significant reduction in group geometric mean mite counts was achieved from their baseline counts as compared to each post-treatment mite count and up until the last mite counts were performed. Efficacy was confirmed following assessment of individual dog reductions in mite counts ≥ 90% and a cure rate based on an assessment of the number and percentage of dogs that had become mite free after two consecutive zero mite counts.

### Translation

Spanish translation of the article is available in Additional file 1.

## Results

### Assessment of mite counts, efficacy, and related study procedures

The GM number of live *Demodex* spp. mites based on skin scrapings and percent reduction for each assessment day are summarized in Table 1. All dogs had severe mite infestations as demonstrated by the high pre-treatment geometric mean baseline counts (mean counts of 631.7 live mites/dog). A reduction in mite numbers exceeding 99% was observed throughout the study, with no mites (100% reduction) being observed on Days 28, 42, 70 and 84. The decrease in mite counts from pre-treatment skin scrapings was statistically significant on Days 28 (*t*_(9)_ = 23.5, *P* < 0.0001), 42 (*t*_(9)_ = 33.3, *P* < 0.0001) and 56 (*t*_(9)_ = 13.69, *P* < 0.0001). No statistical testing was possible on Days 70 and 84 since skin scrapings were performed on only one dog at these time points. In each model the effect for dog was non-significant.

**Table 1:** Group geometric mean (GM) mite count reduction from baseline per skin scraping day in dogs treated with three consecutive monthly oral doses of lotilaner

Day	Baseline GM	GM post-treatment	Percentage reduction
Day 28	631.7	0.0	100
Day 42	631.7	0.0	100
Day 56	631.7	0.3	≥ 99.9
Day 70	631.7	0.0	100
Day 84	631.7	0.0	100

### Individual percentage decrease in mite counts and cure rate

All lotilaner-treated dogs had a 100% reduction in mite numbers by Day 28 based on skin scrapings and for the duration of the assessments, except for one dog where 10 mites were recorded in the skin scrapings on Day 56. All dogs were mite-free at the end of the last two assessment time points (Days 70 and 84), demonstrating 100% clearance of mites following three consecutive monthly treatments with lotilaner.

Based on the hypothesis that the observed cure rate of 100% as seen in this study was no different from a theoretical spontaneous self-cure rate of 50%, this was assessed statistically using the exact test of a binomial proportion. The result of this test showed that the cure rate in this study was significantly greater than 50% (*P* = 0.0002, 95% exact confidence interval: 69.1–100%).

### Clinical signs and symptoms

The occurrence of erythematous papules, crusts, casts or scales on dogs were documented over the 84 day study period. These results are summarized in Table 2.

**Table 2:** The occurrence of clinical signs of demodicosis including erythematous papules, crusts, casts or scales in dogs treated with three consecutive monthly oral doses of lotilaner

Clinical sign	Day ‐2	Day 28	Day 42	Day 56	Day 70	Day 84
Erythematous patches	0	10% (1/10)	0	0	0	0
Crusts, casts or scales	90% (9/10)	30% (3/10)	40% (4/10)	20% (2/10)	20% (2/10)	10% (1/10)

No erythematous patches were visible on the dogs prior to or following treatment, except from one dog on Day 28. The occurrence of crusts, casts or scales was reduced from 90% (9/10) prior to treatment to 20% (2/10) at 8 weeks and 10% (1/10) at 12 weeks following initiation of treatment.

**Fig. 1:**
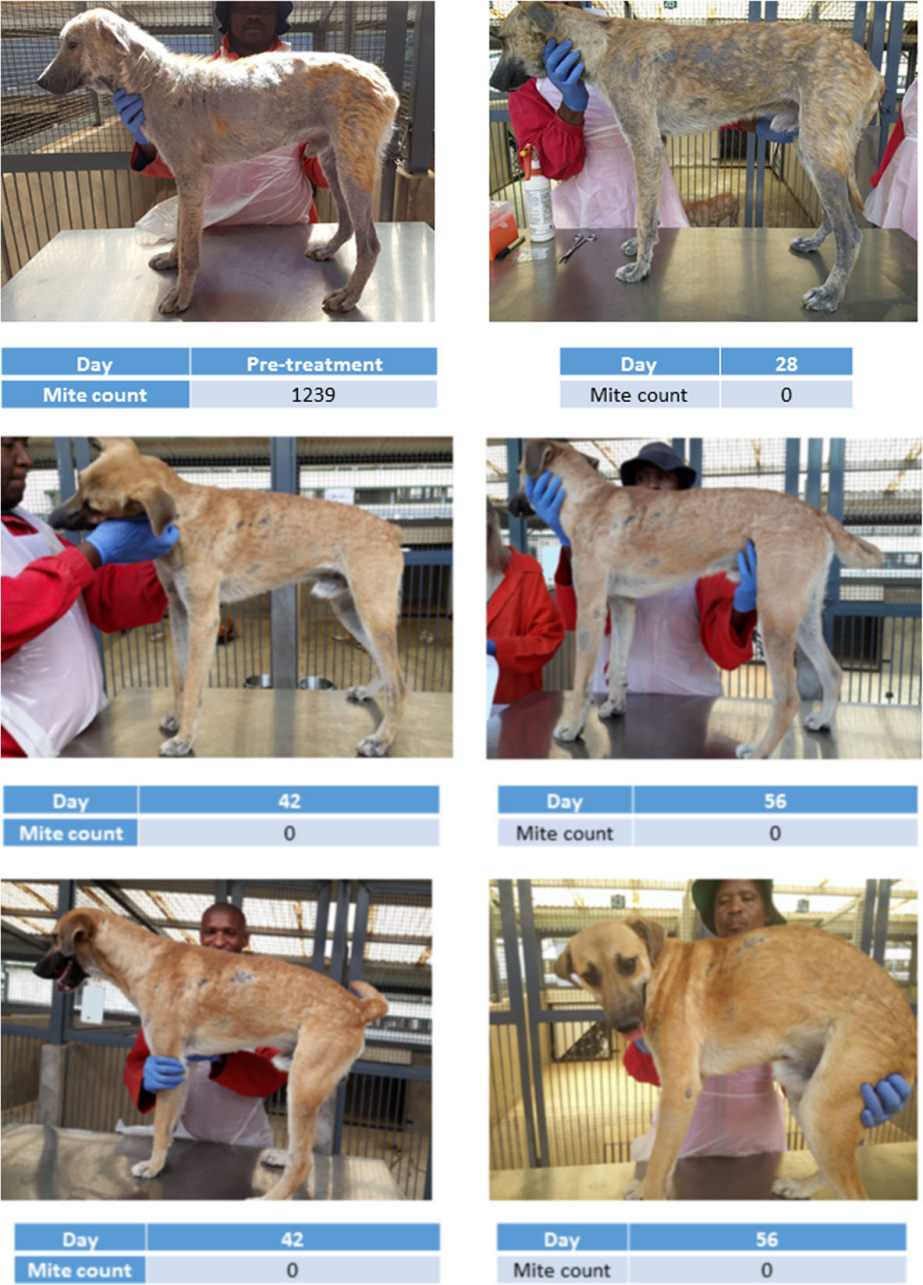
Photographic documentation of hair regrowth in a lotilaner-treated dog (Animal ID 86A95B) with significant hair loss and with high mite counts at baseline as compared to Day 84 post-treatment

Hair re-growth, compared to the proportion of the body area covered by hair prior to treatment, was also assessed during the study. A marked improvement in hair re-growth was observed in all the dogs from 6 weeks following initiation of treatment. An example of hair regrowth and improvement in skin lesions is exemplified in the series of photographs from baseline to Day 84 for one of the treated dogs (Fig. 1).

### Health observations

There were no adverse events in this study that were considered related to treatment with lotilaner.

## Discussion

The single center efficacy study as reported here documents the oral systemic acaricidal efficacy of the novel isoxazoline, lotilaner, against *Demodex* spp. mange mites in dogs. A single oral dose (on Days 0 and 28) at a minimum of 20 mg/kg resulted in 100% reduction in *Demodex* mite counts at 28 and 42 days based on skin scrapings and ≥ 99.9% at the day 56 skin scrapings performed just prior to the third and final dose. No mites were observed (100% reduction) on days 70 and 84. The decrease in mite counts from baseline pre-treatment skin scrapings was statistically significant (*P* < 0.0001). All dogs were mite-free at the end of the last two assessment time points (Days 70 and 84), therefore the cure rate was 100% following three consecutive monthly treatments with lotilaner.

Generalized demodicosis as presented in dogs can be difficult to treat due to the extensive nature of skin lesions and high mite counts when first diagnosed with this skin disease. As a result, available and historical treatment options have generally required repeated applications over long periods and often using off-label elevated dosages of macrocyclic lactones. Adverse effects are not uncommon based on these treatment regimens and can be even more problematic in avermectin sensitive dog breeds [15].

This study was conducted without a negative control group due to animal welfare concerns. Thus, the potential for self-cure in the dogs enrolled in this study cannot be discounted and efficacy of treatments could be overestimated based on this laboratory model and not truly reflect the efficacy seen under clinical field situations. However, all dogs enrolled in this study had severe clinical signs of generalized demodicosis, which is generally considered a chronic disease and, thus, the likelihood of spontaneous self-cure is unlikely to occur and would not resolve without treatment [3]. Spontaneous self-cure of juvenile-onset generalized demodicosis has previously been reported as high as 50% [12]. Although self-cure percentages this high have not been observed for adult-onset or chronic generalized demodicosis, the hypothesis that the observed cure rate of 100% seen in this studywas no different from a theoretical, spontaneous self-cure rate of 50% was assessed. As described in the results section, this was assessed statistically using the exact test of a binomial proportion. The result of this test showed that the cure rate in this study was significantly greater than 50% (*P* = 0.0002; 95% exact confidence interval 69.1%–100%) indicating that the results of this study are not due to spontaneous self-cure and are clearly attributable to treatment with lotilaner. In this study, lotilaner was administered at the minimum dose of 20 mg/kg recommended for month-long control of fleas and ticks and provided effective control of *Demodex* mites as discussed above, with the near eradication (> 99.9%) of mites following the second monthly dose (Day 28) and a 100% cure rate after the third dose. Concurrently with these rapid and significant reductions in mite counts was the rapid resolution of clinical signs, indicating the potential of lotilaner to be part of a treatment regimen that includes convenient monthly oral dosing for generalized demodicosis in dogs. In future studies, the promising results as presented herein using lotilaner will be confirmed in multicentric studies using client owned dogs presenting with generalized demodicosis. The miticidal efficacy of other isoxazoline containing drug products have demonstrated similar or slightly less efficacy after consecutive monthly oral administrations in dogs with generalized demodicosis [16, 17].

There were no adverse reactions to treatment with oral lotilaner in the present study.

## Conclusions

It can be concluded that lotilaner was effective in successfully treating generalized demodicosis in dogs after three consecutive monthly treatments at a minimum dose of 20 mg/kg. This was demonstrated by significant (*P* < 0.0001) differences between baseline and each of the post-treatment skin scraping assessment days, where efficacies exceeding 99% and up to 100% were obtained throughout the study. Additionally, all dogs were mite-free at the end of the last two assessment time points (Days 70 and 84), therefore the cure rate was 100% following three consecutive monthly treatments with lotilaner. There were also significant improvements in the clinical condition of all lotilaner-treated dogs from baseline to Day 84 as seen in the assessment of measured clinical scores.

## Additional file

Additional file 1: Spanish translation of the article. (PDF 134 kb)
