# Supplementary material for: Efficacy of lotilaner (Credelio™), a novel oral isoxazoline against naturally occurring mange mite infestations in dogs caused by Demodex spp
Source: Parasit Vectors. 2017 Nov 1;10:532. doi: 10.1186/s13071-017-2472-2 (PMC5664441; doi:10.1186/s13071-017-2472-2)
Supplement: Additional file 1: — Spanish translation of the article. (PDF 134 kb) [file 13071_2017_2472_MOESM1_ESM.pdf]

Eficacia de lotilaner (Credelio™), una nueva isoxazolina oral contra infestaciones de sarna presentes de manera natural en perros causadas por *Demodex* spp.

Daniel E. Snyder[1*], Scott Wiseman[2] y Julian E. Liebenberg[3]

[1]Elanco Animal Health, Investigación y Desarrollo, 2500 Innovation Way, Greenfield, IN 46140, EUA
[2]Elanco Animal Health, Basingstoke, Hampshire, RG24 9NL, RU
[3]ClinVet International (pty) Ltd, Uitsigweg, Bainsvlei 9338, Bloemfontein, República de Sudáfrica

*Correspondencia: snyder_daniel_e@elanco.com

Direcciones de correo electrónico de los autores:
snyder_daniel_e@elanco.com
wiseman_scott@elanco.com
Julian.Liebenberg@clinvet.com

Resumen

Antecedentes: La eficacia oral sistémica de lotilaner (Credelio™, Elanco) fue evaluada contra el *Demodex* spp. en perros infestados naturalmente con demodicosis generalizada.

Métodos: En este estudio, 10 perros con signos clínicos de demodicosis generalizada y positivos a ácaros *Demodex* spp. basados en raspados de piel se asignaron a un solo grupo tratado oralmente con lotilaner (dosis mínima de 20 mg/kg) en los Días 0, 28 y 56.

Resultados: Para los perros tratados con lotilaner, los conteos de ácaros pretratamiento realizados con raspados de piel y llevados a cabo en cinco diferentes sitios se redujeron en > 99.9% ($P < 0.0001$) hasta 56 días después de la primera y segunda dosis mensual. No se detectaron ácaros vivos después del Día 56 hasta e incluyendo el Día 84 post-tratamiento con un 100% de eficacia en cada perro con infestación por el ácaro *Demodex*. Nueve de 10 perros estaban libres de ácaros en el Día 28 (primera evaluación) hasta el Día 84 (final del estudio) y los ácaros vivos solamente se

encontraron una vez en un perro (Día 56) después del tratamiento de lotilaner. Todos los perros en el grupo tratado con lotilaner mostraron una mejoría marcada en los signos clínicos de demodicosis y no hubieron eventos adversos asociados con el fármaco. Se observa una marcada mejoría en recrecimiento del pelo en todos los perros desde las 6 semanas después de iniciado el tratamiento.
Conclusiones: En este estudio lotilaner administrado a una dosis oral mínima de 20 mg/kg resultó altamente efectivo al reducir y eliminar los conteos de ácaros vivos en peros con infestaciones naturales de *Demodex* spp.
Palabras clave: Credelio™, *Demodex* spp., Demodicosis, Perro, Lotilaner, Sarna, Oral

Antecedentes

La demodicosis canina es una enfermedad parasitaria de la piel caracterizada por un aumento excesivo de ácaros *Demodex* spp. dentro de las glándulas pilocebaceas [1]. Están implicadas tres especies de ácaros *Demodex*, *Demodex canis* siendo el más común [2]. La demodicosis canina se clasifica como localizada o generalizada de acuerdo al grado de enfermedad. La demodicosis localizada es una enfermedad benigna y en muchos casos se resuelve espontáneamente al cabo de 6 a 8 semanas [1]. La demodicosis se considera generalizada cuando cinco o más áreas de enfermedad localizada se observan, pododemodicosis observada en dos o más patas, o cuando toda una región del cuerpo está involucrada. La demodicosis también puede categorizarse como juvenil (perros hasta 18 meses de edad), brote en adultos (perros generalmente mayores de 4 años sin una historia previa de la enfermedad), o crónica generalizada (enfermedad persistente durante al menos 6 meses) [2, 3].

La demodicosis crónica generalizada es una enfermedad de la piel frustrante y difícil de tratar [4, 5]. En estos perros con demodicosis crónica generalizada a largo plazo, no es probable que la enfermedad se resuelva sin terapia [3]. Actualmente, las opciones terapéuticas disponibles involucran períodos de tratamiento diario, semanal, bi-semanal o mensual por períodos de 3 meses o más [1, 5, 6].

Lotilaner (Credelio™, Elanco) es una nueva isoxazolina con una actividad prolongada potente demostrada contra garrapatas y pulgas [7, 8] después de la administración oral. El estudio aquí reportado se condujo para evaluar la eficacia y la seguridad de lotilaner contra las infestaciones naturales de *Demodex* spp. en perros con demodicosis generalizada a una dosis mínima de 20 mg/kg como se propone para la administración mensual para el tratamiento y el control de pulgas y garrapatas.

Métodos

Este estudio fue conducido en la República de Sudáfrica en cumplimiento con las Buenas Prácticas Clínicas [9] y las guías locales de bienestar animal [10]. El protocolo del estudio fue revisado y aprobado por los Comités Institucionales de Cuidado Animal y Uso de ClinVet y Elanco.

Animales y alojamiento

Los perros se alojaron individualmente en jaulas bajo estrictas condiciones de cuarentena durante todo el estudio. No era posible el contacto físico entre los perros, pero si tenían contacto visual y auditivo con conespecíficos y las condiciones de alojamiento iban de acuerdo con las aceptadas en las guías de bienestar. Cada perro fue identificado individualmente por un implante de microchip alfanumérico. Los perros incluidos en este estudio no fueron tratados con un ectoparasiticida por al menos 90 días y contaban con buena salud en el momento del tratamiento excepto por los signos de alopecia y lesiones en la piel asociadas a la demodicosis generalizada. Los perros fueron alimentados con una ración de mantenimiento apropiada de una dieta comercial canina concentrada durante todo el estudio. El agua estuvo disponible *ad libitum*.

El estudio incluyó 10 perros mestizos (7 machos y 3 hembras), obtenidos localmente, de ≥ 6 meses de edad y pesando de 9.59 a 17.65 kg. Los perros fueron incluidos en el estudio basándose en los raspados positivos de la piel con las infestaciones naturales de *Demodex*. Todos los perros incluidos exhibieron signos clínicos de demodicosis generalizada incluyendo lesiones en piel como alopecia, eritema, comedones, pápulas, pústulas, escoriaciones, descamación o costras involucrando toda una región del cuerpo o cinco o más lesiones localizadas en áreas discretas (cada una con un diámetro > 2.5 cm), o pododemodicosis involucrando dos o más patas, y la presencia confirmada de ácaros *Demodex* vivos basado en los raspados profundos de piel [11, 12]. Adicionalmente, ningún perro había recibido corticosteroids de corta acción al cabo de una semana o corticosteroides de larga acción al cabo de cuatro semanas del Día 0.

Diseño experimental general y métodos

Los perros fueron aclimatizados a las condiciones del sitio del estudio durante 7 días previos al tratamiento. Este estudio se condujo con un grupo control negativo debido a las preocupaciones de bienestar animal. El Día 0 se definió como el día cuando todos los 10 perros recibieron su primer tratamiento. Se llevó a cabo un examen físico en cada perro por el veterinario tratante en el Día -7

para determinar la salud y adecuación previo a la inclusión en el estudio, y se hicieron las observaciones de salud general al menos una vez al día a lo largo del estudio. Adicionalmente, se condujo un examen clínico en cada perro en los Días -2, 14, 28, 42, 56, 70 y 84.

Comenzando en el Día -7 todos los perros incluidos recibieron un antibiótico apropiado (como lo prescribió el veterinario tratante) para pioderma. Concurrentemente, con la terapia con antibiótico, todos los perros recibieron un destilado de levadura diario para apoyar su flora intestinal. En los Días -7 y 27, se tomaron biopsias de cada perro bajo sedación con un sedante adecuado, y una terapia antimicrobiana concomitante se continuó hasta que se recibieron los resultados de estas biopsias. Basándose en los resultados de la biopsia del Día 27, sí no habían células inflamatorias o bacterias presentes, la terapia antimicrobiana se descontinuaba. Basándose en los resultados de la biopsia del Día 27, sí todavía había evidencia de infección activa, pero menos células inflamatorias y/o bacterias en comparación con las primeras biopsias de piel, el antibiótico y el destilado de levadura originalmente utlizado se continuaban por otras cuatro semanas. Sí se detectaba deterioro clínico y citológico basado en los resultados de la biopsia del Día 27, la terapia antibacteriana y el destilado de levadura se continuaban con un antibiótico alternativo como lo prescribió el veterinario tratante.

Lotilaner fue administrado una vez en los Días 0, 28, y 56 y se basó en los pesos de los perros, redondeado al 0.1kg más cercano, registrados en el día anterior a cada tratamiento. El conteo de ácaros se llevó a cabo en los días de dosificación previo a que cada perro fuera dosificado con lotilaner. Los pesos corporales obtenidos durante la aclimatización se utilizaron para la dosificación del Día 0 y el peso corporal medido en el día anterior a cada tiempo de dosificación programada (Días 28 y 56) se utilizaron para determinar la fuerza apropiada y el número de tabletas de lotilaner que se iban a administrar en cada tiempo de dosificación, respectivamente.

La dosificación oral se condujo en estado alimentado. En el día previo a cada tiempo de dosificación programado (Día -1, Día 27 y Día 55), a cada perro se le ofreció solamente la mitad de su ración diaria de alimento. En cada día de tratamiento (Días 0, 28 y 56), 30 ± 5 min previo a cada tratamiento de lotilaner programado, a cada perro se le ofreció la ración diaria de alimento enlatado húmedo a la dosis recomendada. El tiempo de alimentación se registró y en el momento del tratamiento programado (± 5 min), se les retiró el alimento residual, se pesó y se registró. Al menos 1/3 de la ración diaria ofrecida se requirió para ser consumida por el perro antes de la administración de las tabletas de lotilaner. Sí el perro no consumía alimento húmedo después de 15 min (± 5 min), entonces se alimentaba manualmente colocando pequeñas cantidades de alimento en la parte

posterior de su boca y permitiendo que el perro lo tragara hasta que la mitad de la lata de alimento húmedo, aproximadamente, hubiera sido consumida y luego se procedía al tratamiento como se había programado. Las tabletas se administraron oralmente por tableta para asegurar una dosificación precisa y completa. Cada perro fue observado por varios minutos post-dosificación, y luego aproximadamente una hora de intervalo después de la dosificación hasta 4 horas post-dosificación se observó a cada perro para determinar cualquier signo anormal de salud.

Para evitar cualquier problema de contaminación de los perros durante el conteo de ácaros y los exámenes clínicos, el personal del estudio se cambiaba la ropa protectora entre perros, utilizaba guantes y equipo por separado con cada perro, y limpiaban la superficie de la mesa de auscultación utilizada para el raspado y/o conteo de ácaros.

Todos los perros fueron examinados para determinar signos clínicos de demodicosis (incluyendo documentación fotográfica) y los raspados en piel/conteo de ácaros se llevaron a cabo en los Días -2, 28, 42, 56, 70 y 84. Sí cualquier perro demostraba tener dos conteos consecutivos de ácaros vivos negativos (cero) en o después del Día 56, los raspados profundos de piel adicionales en los tiempos listados anteriormente se detenían por razones de bienestar animal debido a la naturaleza invasiva del procedimiento, pero las evaluaciones clínicas y la documentación fotográfica se continuaron hasta el Día 84 para evaluar totalmente la resolución de las lesiones en piel. Las infestaciones de ácaros se evaluaron usando los raspados profundos en piel tomados de cinco sitios diferentes en cada perro en cada tiempo programado mostrando la evidencia clínica más severa de una infestación activa de ácaros basada en el examen de piel visiblemente afectada con lesiones. El material del raspado se transfería a una laminilla, se mezclaba con aceite mineral y se examinaba bajo el microscopio usando un aumento de 40× o 100× para contar a los ácaros adultos y a los inmaduros. Los signos clínicos y el grado de lesiones demodé ticas en cada perro se evaluaron en los días durante los cuáles los raspados se hicieron y se registraron en una forma estandarizada. Los siguientes parámetros se evaluaron para cada perro y se dibujó una silueta (del lado izquierdo y derecho) de un perro: (i) áreas del cuerpo cubiertas de escoriaciones, descamación y costras; (ii) áreas del cuerpo con pérdida de pelo (1, ligero adelgazamiento del pelo; 2, pérdida conspicua de pelo; 3, sin pelo); y (iii) áreas del cuerpo con eritema. Los signos clínicos de demodicosis generalizada se evaluaron como un porcentaje de superficie corporal afectada por las lesiones de la piel seguidas de la asignación de una calificación clínica para cada uno de los cuatro parámetros: (i) comedones, pústulas y pápulas; (ii) escoriaciones, costras y descamación; (iii) alopecia; y (iv) eritema.

Conteo de ácaros y evaluación de eficacia

La variable de evaluación primaria en este estudio fue la diminución del porcentaje en el conteo de ácaros (ácaros vivos inmaduros y adultos combinados) comparándolos con los conteos base de los días 28, 42, 56, 70 y 84, después de la administración mensual de lotilaner.

Se calculó la eficacia usando medias geométricas con fórmula de Abbott:

Eficacia (%) = (Mpre – Mpost) / Mpre × 100

en donde Mpre fue el número promedio del grupo de los conteos de ácaros pre-tratamiento, y Mpost el número medio del grupo de los conteos de ácaros post-tratamiento.

En donde no hubo conteos disponibles para un perro en o después del día 56, porque el perro tuvo dos conteos sucesivos de cero en puntos en el tiempo anteriores, luego se usó cero en la evaluación.

Adicionalmente, una variable de evaluación secundaria de la eficacia fue la disminución en el porcentaje individual del conteo de ácaros pre-administración al conteo de ácaros post-administración en cada perro o en cada día de evaluación y se calculó por:

Disminución % (individual) = (Pre-administración – Post-administración)/Pre-administración × 100

en donde, el conteo pre-administración es el conteo de ácaros pre-administración de un perro y post-administración es el conteo de ácaros post-administración de un perro.

El número de ácaros en cada día de evaluación y el porcentaje de reducción en el conteo de ácaros se tabularon, con la siguiente estadística descriptiva: media, desviación estándar (DE), media geométrica (MG), mínimo y máximo. Adicionalmente, otra variable de evaluación secundaria de eficacia fue la tasa de curación, definida a continuación:

Tasa de curación (expresada como un porcentaje) = número de perros libres de ácaros después de dos conteos consecutivos de ácaros post-tratamiento / número total de perros con conteos de ácaros pre-tratamiento.

Signos clínicos

Los datos registrados durante las evaluaciones clínicas en las escoriaciones, descamaciones, costras y áreas de pérdida de pelo y eritema fueron resumidas por el investigador. Los cambios general en la apariencia clínica también fueron documentados por medio de las fotografías pre- y post-administración de cada perro. Este registro fotográfico se utilizó para mostrar el grado general y la resolución de las lesiones demodécicas de cada perro.

Más aún, el número de perros afectados por el eritema, escoriaciones, descamaciones y costras fue descrito para determinar los días de evaluación pre-administración y los días diferentes para la evaluación post-administración. Una evaluación semi-cuantitativa de re-crecimiento también fue hecha y una calificación se otorgó para cada perro en los diferentes días de evaluación post-dosificación.

Se asignó una evaluación semi-cuantitativa del re-crecimiento del pelo: (i) si las áreas corporales con re-crecimiento 0-50% comparado con lo registrado durante la evaluación pre-administración; (ii) áreas corporales con re-crecimiento estimado > 50% ≤ 90% en comparación con lo registrado en la evaluación pre-administración; y (iii) áreas corporales con re-crecimiento de pelo estimado en > 90% en comparación con lo registrado durante la evaluación pre-administración.

Los signos clínicos se analizaron descriptivamente usando los conteos de frecuencia y porcentajes para evaluar la eficacia de las administraciones mensuales de lotilaner.

Métodos estadísticos

La unidad experimental fue el perro individual. Las guías de la WAAVP [13] y las guías de EMEA [14] recomiendan un mínimo de seis sujetos por grupo para usarlos en estudios que evalúan la eficacia de los productos contra pulgas y garrapatas. Con un mínimo de cinco perros en el grupo de tratamiento de lotilaner, fue posible detector una baja significativa estadísticamente (a un nivel de error tipo I del 5%) de > 90% en los conteos de ácaros con más de 90% de poder. El uso de 10 perros se consideró apropiado para proveer resultados estadísticos robustos y resultados clínicos en este único grupo de estudio. Las pruebas estadísticas se condujeron a un nivel del 5% de significancia.

Se investigó el efecto del tratamiento sobre el conteo de ácaros para cada perro comparando la base del pre-tratamiento y los conteos de ácaros post-tratamiento en un modelo de ANOVA con efectos de tiempo y perro (pre- o post-tratamiento). Se hicieron coincidir modelos separados para los Días de Estudio 28, 42, y 56. Solamente a un perro se le realizaron los raspados en piel en los Días 70 y 84, por lo que no fue posible hacer pruebas estadísticas.

Comparación de conteos de ácaros

Los conteos de ácaros en la base del pre-tratamiento y el post-tratamiento para cada perro se compararon por un modelo de ANOVA con efectos de tiempo y perro (pre- o post-tratamiento) y los efectos de perro usando SAS Versión 9.3 TS Nivel 1M2 (SAS Institute Inc., Cary, NC, EUA). Para evaluar la hipótesis que el porcentaje observado de la tasa de curación calculada en este estudio no era diferente de la tasa de auto-curación espontánea del 50%, se llevó a cabo un análisis estadístico usando la prueba exacta de proporción binomial.

Indicación de eficacia

La eficacia se indicó para lotilaner contra los ácaros si se lograba una reducción de ≥ 90% estadísticamente significativa en la media geométrica del grupo de los conteos de ácaros con respecto a sus conteos base comparados con cada conteo de ácaros post-tratamiento y hasta que se llevara a cabo el último conteo de ácaros. Se confirma la eficacia después de la evaluación de las reducciones en el conteo de ácaros por perro individualmente ≥ 90% y la tasa de curación se basó en una evaluación en el número y el porcentaje de perros que se convirtieran en libres después de dos conteos consecutivos de ácaros en cero.

Resultados

La evaluación de los conteos de ácaros, la eficacia y los procedimientos relativos al estudio

El número de la MG de los ácaros vivos de *Demodex* spp. basados en los raspados de piel y el porcentaje de reducción para cada día de evaluación se resumen en el Tabla 1. Todos los perros tuvieron severas infestaciones de ácaros como lo demuestra la elevada media geométrica de los conteos base (media de los conteos de 631.7 ácaros vivos/perro). Se observó una reducción en los números de ácaros que exceden a un 99% a lo largo del estudio sin ácaros (100% reducción) siendo observados en los Días 28, 42, 70 y 84. La disminución en los conteos de ácaros de los raspados en piel pre-tratamiento fueron significativos estadísticamente en los Días Días 28 ($t_{(9)} = 23.5$, $P < 0.0001$), 42 ($t_{(9)} = 33.3$, $P < 0.0001$), y 56 ($t_{(9)} = 13.69$, $P < 0.0001$). No fue posible realizar pruebas estadísticas en los Días 70 y 84 ya que los raspados de piel se llevaron a cabo en un solo perro en esos puntos de tiempo. En cada modelo el efecto por perro no fue significativo.

Disminución del porcentaje individual en el conteo de ácaros y la tasa de curación

Todos los perros tratados con lotilaner tuvieron una reducción del 100% en los números de ácaros para el Día 28 basada en los raspados de piel y para la duración de las evaluaciones, excepto para un perro se registraron 10 ácaros en los raspados de piel en el Día 56. Todos los perros estaban libres de ácaros al final de los dos últimos puntos de tiempo de evaluación (Días 70 y 84), demostrando una eliminación del 100% de ácaros después de tres tratamientos mensuales consecutivos con lotilaner.

Basado en la hipótesis de que la tasa de curación observada era del 100%, como se ve en este estudio, y no resultó diferente de la teoría de la tasa de auto-curación espontánea del 50%, esto se evaluó estadísticamente usando la prueba exacta de la proporción binomial. El resultado de esta prueba mostró que la tasa de curación en este estudio fue significativamente mayor al 50% (P = 0.0002, 95% intervalo exacto de confianza: 69.1–100%).

Signos clínicos y síntomas

La presencia de pápulas eritematosas, costras, escoriaciones o descamación en perros se documento durante los 84 días del período de estudio. Estos resultados se resumen en el Tabla 2.

No había parches eritematosos visibles en los perros antes o después del tratamiento, excepto por un perro en el Día 28. La presencia de costras, escoriaciones, o descamaciones se redujo del 90% (9/10) previo al tratamiento a un 20% (2/10) a las 8 semanas y un 10% (1/10) a las 12 semanas después del inicio del tratamiento.

El re-crecimiento del pelo, en comparación con la proporción del área del cuerpo cubierta por pelo previo al tratamiento también fue evaluado durante el estudio. Una mejoría marcada en el re-crecimiento del pelo se observó en todos los perros a las 6 semanas después del inicio del tratamiento. Un ejemplo del re-crecimiento del pelo y la mejoría de las lesiones en piel se ejemplifica en la serie de fotografías de la base al Día 84 para uno de los perros tratados (Fig. 1).

Observaciones de salud

No hubo eventos adversos en este estudio que se relacionara con el tratamiento con lotilaner.

Discusión

El único estudio de eficacia central reportado aquí documenta la eficacia acaricida, oral sistémica de la nueva isoxazolina, lotilaner, contra los ácaros de la sarna, *Demodex* spp. Una sola dosis oral (en

los Días 0 y 28) a un mínimo de 20 mg/kg resultó en un 100% de reducción en los conteos de los ácaros *Demodex* a los 28 y 42 días basados en los raspados en piel y ≥ 99.9% en el día 56 se llevaron a cabo los raspados en piel justo antes de la tercera dosis y la dosis final. No se observaron ácaros (100% reducción) en los días 70 y 84. La disminución en los conteos de ácaros en los raspados en piel de la base del pre-tratamiento fueron significativos estadísticamente ($P < 0.0001$). Todos los perros estaban libres de ácaros al final de los últimos puntos de tiempo de evaluación (Días 70 y 84), por lo tanto, la tasa de curación fue del 100% después de tres tratamientos mensuales consecutivos con lotilaner.

Cuando la demodicosis generalizada se presenta en perros puede ser difícil de tratar debido a la naturaleza extensiva de las lesiones en piel y los conteos de ácaros altos cuando se diagnostica por primera vez esta enfermedad de la piel. Como resultado, las opciones disponibles e históricas de tratamiento generalmente requieren de aplicaciones repetidas en períodos prolongados y con frecuencia usando dosis elevadas fuera-de-etiqueta de lactonas macrocíclicas. Los efectos adversos no son comunes basándose en estos regímenes de tratamiento y pueden ser aún más problemáticos en razas de perros sensibles a la avermectina [15].

Este estudio se condujo sin un grupo control negativo debido a las preocupaciones de bienestar animal. Por ende, el potencial para la auto-curación en los perros incluidos en el estudio no puede descartarse y la eficacia de los tratamientos podría estar sobrestimada basada en este modelo de laboratorio y no refleja verdaderamente la eficacia vista bajo situaciones clínicas de campo. Sin embargo, todos los perros incluidos en este estudio tuvieron severos signos clínicos de demodicosis generalizada, que generalmente, se considera una enfermedad crónica y, por ende, la probabilidad de una auto-curación espontánea es poco probable que ocurra y no se resolverá sin tratamiento [3]. La auto-curación espontánea de un brote juvenil generalizado de demodicosis había sido reportada previamente, siendo tan alta como un 50% [12]. Aunque los porcentajes de auto-curación tan altos como este no han sido observados para un brote en adultos o una demodicosis crónica generalizada, la hipótesis de que la tasa de curación observada sea del 100% como se vio en este estudio, se evaluó que una tasa teórica de autocuración espontánea del 50% no fue diferente. Como ya se describió en la sección de resultados, esto se evaluó estadísticamente usando la prueba exacta de proporción binomial. El resultado de esta prueba mostró que la tasa de curación en este estudio fue significativamente mayor que el 50% ($P = 0.0002$; 95% el intervalo exacto de confianza 69.1%-100%) indicando que los resultados de este estudio no se deben a la auto-curación espontánea y son claramente atribuibles al tratamiento con lotilaner. En este estudio, lotilaner se

administró a la dosis mínima de 20 mg/kg recomendado para el control de pulgas y garrapatas por un mes y proporcionó un control efectivo de ácaros de *Demodex* como se discutió anteriormente, con la casi erradicación de los ácaros (> 99.9%) de la segunda dosis mensual (Día 28) y una tasa de curación del 100% después de la tercera dosis. Concurrentemente, con estas reducciones rápidas y significativas en los conteos de ácaros fue la rápida resolución de los signos clínicos, indicando el potencial de lotilaner para ser parte de un régimen de tratamiento que incluye una dosis oral mensual conveniente para la demodicosis generalizada en perros. En estudios futuros, los resultados prometedores como se presentan aquí usando lotilaner serán confirmados en estudios multicéntricos usando perros con dueño que presenten una demodicosis generalizada. La eficacia acaricida de otros productos farmacéuticos que contienen isoxazolina han demostrado similar o ligeramente menor eficacia después de las administraciones orales mensuales consecutivas en los perros con demodicosis generalizada [16, 17].

No hubo reacciones adversas al tratamiento con lotilaner oral en este estudio.

Conclusiones

Puede concluirse que lotilaner fue efectivo en perros con demodicosis generalizada tratados exitosamente después de tres tratamientos mensuales consecutivos a una dosis mínima de 20 mg/kg. Esto fue demostrado por las diferencias significativas ($P < 0.0001$) entre la base y cada uno de los días de evaluación de los raspados de piel post-tratamiento, en donde las eficacias obtenidas excedieron al 99% y hasta el 100% a lo largo del estudio. Adicionalmente, todos los perros estaban libres de ácaros al final de las evaluaciones en los últimos dos puntos de tiempo (Días 70 y 84), por lo tanto la tasa de curación fue del 100% después de los tres tratamientos mensuales consecutivos con lotilaner. También hubieron mejorías significativas en la condición clínica de todos los perros tratados con lotilaner de la base al Día 84 como se vio en la evaluación de las calificaciones clínicas medidas.

Reconocimientos

Los autores reconocen al personal del estudio de ClinVet International por sus contribuciones en la colección de datos para este estudio.

Fondos

Todos los estudios fueron patrocinados por Elanco.

Disponibilidad de datos y materiales

Los grupos de datos que apoyan las conclusiones de este artículo se incluyeron dentro del artículo.

Contribuciones de los autores

DES, SW y JEL apoyaron con el diseño y conducción de los estudios, interpretación de los datos y revisión del manuscrito. Todos los autores leyeron y aprobaron el manuscrito final.

Intereses competentes

DES y SW son empleados de Elanco. JEL (investigador) fue contratado para estos estudios a través de ClinVet International.

Consentimiento para la publicación

No aplicable.

Aprobación de ética

El protocolo del estudio fue aprobado para el sitio del estudio por el Comité Institucional de Cuidado Animal y Uso; CV 15/225 – 14 Diciembre 2015.

Referencias

1. Paradis M. New approaches to the treatment of canine demodicosis. Vet Clin North Am Small Anim Pract. 1999;29:1425-36.

2. Shipstone M. Generalized demodicosis in dogs, clinical perspective. Aust Vet J. 2000;78:240-2.

3. Paradis M, Page N. Topical (pour-on) ivermectin in the treatment of chronic generalized demodicosis in dogs. Vet Dermatol. 1998;9:55-59.

4. Medleau L, Ristic, Z. Treating chronic refractory demodicosis in dogs. Vet Med. 1994;89:775-77.

5. Miller WH, Scott DW, Cayatte M, Buerger RG, Bagladi MS. Clinical efficacy of increased dosages of milbemycin oxime for treatment of generalized demodicosis in adult dogs. JAVMA. 1995;207:1581-84.

6. Wagner R, Wendlberger U. Field efficacy of moxidectin in dogs and rabbits naturally infested with *Sarcoptes* spp., *Demodex* spp. and *Psoroptes* spp. mites. Vet Parasitol. 2000;93:149-58.

7. Cavalleri D, Murphy M, Seewald W, Drake J, Nanchen S. A randomized, blinded, controlled field study to assess the efficacy and safety of lotilaner tablets (Credelio™) in controlling fleas in client-owned dogs in European countries. Parasit Vectors. 2017 (In press).

8. Murphy M, Garcia R, Karadzovska D, Cavalleri D, Snyder DE, Seewald W, Real T, Drake J, Wiseman S, Nanchen S. Laboratory evaluations of the immediate and sustained efficacy of lotilaner (Credelio™) against four common species of ticks affecting dogs in North America. Parasit Vectors. 2017; (In press).

9. EMEA. Guideline on good clinical practices. VICH Topic GL9. http://www.ema.europa.eu/docs/en_GB/document_library/Scientific_guideline/2009/10/WC500004343.pdf. Accessed 05 Jun 2017.

10. South African National Standard: The care and use of animals for scientific purposes. (SANS10386:2008)

11 Mueller RS, Bensignor E, Ferrer L, Holm B, Lemarie S, Paradis M, Shipstone A. Treatment of demodicosis in dogs: 2011 clinical practice guidelines. Vet Dermatol. 2012;23: 86–96.

12 Muller G, Kirk R. Parasitic skin disease. In: MillerWH, Griffin CE, Campbell KL, editors. Small Animal Dermatology. 7th ed. St. Louis: Saunders Elsevier; 2013. p. 304-313.

13 Marchiondo AA, Holdsworth PA, Fourie LJ, Rugg D, Hellmann K, Snyder DE, Dryden MW. World Association for the Advancement of Veterinary Parasitology (W.A.A.V.P.) second edition: Guidelines for evaluating the efficacy of parasiticides for the treatment, prevention and control of flea and tick infestations on dogs and cats. Vet. Parasitol. 2013;1941:84–7.

14 EMEA. "Guidelines for the testing and evaluation of the efficacy of antiparasitic substances for the treatment and prevention of tick and flea infestation in dogs and cats” adopted on 7 November 2007 by the Committee for Veterinary Medicinal Product of the European Agency for the Evaluation of Medicinal Products (EMEA/CVMP/005/2000-Rev.2).

15 Merola VM, Eubig PA. Toxicology of avermectins and milbemycins (macrocyclic lactones) and the role of P-glycoprotein in dogs and cats. Vet Clin North Am Small Anim Pract. 2012;42:313-33.

16 Fourie JJ, Liebenberg JE, Horak IJ, Taenzier J, Heckeroth AR, Frénais R. Efficacy of orally administered fluralaner (Bravecto™) or topically applied imidacloprid/moxidectin (Advocate®) against generalized demodicosis in dogs. Parasit Vectors 2015;8:187-97.

17 Six R H, Becskei C, Mazaleski MM, Fourie JJ, Mahabir, SP, Myers M R, Slootman, N. Efficacy of sarolaner, a novel oral isoxazoline, against two common mite infestations in dogs: *Demodex* spp. and *Otodectes cynotis*. Vet Parasitol. 2016;222: 62-66.

Leyenda de la figura

Fig. 1 Documentación fotográfica del re-crecimiento del pelo en un perro tratado con lotilaner (Animal ID 86A95B) con la pérdida de pelo significativa y con los conteos altos de ácaros en la base en comparación con el Día 84 post-tratamiento

Tabla 1 Media geométrica (MG) del grupo en la reducción del conteo de ácaros de la base por día de raspado de piel en perros tratados con tres dosis orales mensuales consecutivas de lotilaner

Día	Base MG	MG post-tratamiento	Porcentaje de reducción
Día 28	631.7	0.0	100
Día 42	631.7	0.0	100
Día 56	631.7	0.3	≥ 99.9
Día 70	631.7	0.0	100
Día 84	631.7	0.0	100

Tabla 2 La presencia de signos clínicos de demodicosis incluyendo pápulas eritomatosas, costras, escoriaciones o descamaciones en perros tratados con tres dosis orales mensuales consecutivas de lotilaner

Signo clínico	Día -2	Día 28	Día 42	Día 56	Día 70	Día 84
Parches eritematosos	0	10% (1/10)	0	0	0	0
Costras, escoriaciones o descamaciones	90% (9/10)	30% (3/10)	40% (4/10)	20% (2/10)	20% (2/10)	10% (1/10)
